# Extended Anticoagulant and Aspirin Treatment for the Secondary Prevention of Thromboembolic Disease: A Systematic Review and Meta-Analysis

**DOI:** 10.1371/journal.pone.0143252

**Published:** 2015-11-20

**Authors:** Paul E. Marik, Rodrigo Cavallazzi

**Affiliations:** 1 Division of Pulmonary and Critical Care Medicine, Eastern Virginia Medical School, Norfolk, Virginia, United States of America; 2 Division of Pulmonary, Critical Care and Sleep Medicine, University of Louisville, Louisville, Kentucky, United States of America; Ottawa Hospital Research Institute, CANADA

## Abstract

**Background:**

Patients who have had an unprovoked deep venous thrombosis (DVT) or pulmonary embolus (PE) are at a high risk for recurrent venous thromboembolism (VTE). Extended “life-long” anticoagulation has been recommended in these patients. However, the risk benefit ratio of this approach is controversial and the role of the direct oral anticoagulants (DOACs) and aspirin is unclear. Furthermore, in some patients with a “weak provoking factor” there is clinical equipoise regarding continuation or cessation of anticoagulant therapy after treatment of the acute VTE event.

**Objective:**

A systematic review and meta-analysis to determine the risks (major bleeding) and benefits (recurrent VTE and mortality) of extended anticoagulation with vitamin k antagonists (VKA), DOACs and aspirin in patients with an unprovoked VTE and in those patients with clinical equipoise regarding continuation or cessation of anticoagulant therapy. In addition, we sought to determine the risk of recurrent VTE events once extended anti-thrombotic therapy was discontinued.

**Data Sources:**

MEDLINE, Cochrane Register of Controlled Trial***s*,** citation review of relevant primary and review articles.

**Study Selection:**

Randomized placebo-controlled trials (RCTs) that compared the risk of recurrent VTE in patients with an unprovoked DVT or PE who had been treated for at least 3 months with a VKA or a DOAC and were then randomized to receive an oral anti-thrombotic agent or placebo for at least 6 additional months. We included studies that included patients in whom clinical equipoise existed regarding the continuation or cessation of anticoagulant therapy.

**Data Extraction:**

Independent extraction of articles by both authors using predefined data fields, including study quality indicators. Data were abstracted on study size, study setting, initial event (DVT or PE), percentage of patients where the initial VTE event was unprovoked, the number of recurrent VTE events, major bleeds and mortality during the period of extended anticoagulation in the active treatment and placebo arms. In addition, we recorded the event rate once extended treatment was stopped. Meta-analytic techniques were used to summarize the data. Studies were grouped according to the type of anti-thrombotic agent.

**Data Synthesis:**

Seven studies which enrolled 6778 patients met our inclusion criteria; two studies evaluated the extended use of Coumadin, three studies evaluated a DOAC and two studies evaluated the use of aspirin. The duration of followup varied from 6 to 37 months. In the Coumadin and aspirin studies 100% of the randomized patients had an unprovoked VTE, while in the DOAC studies between 73.5% and 93.2% of the VTE events were unprovoked. In the control group recurrent VTE occurred in 9.7% of patients compared to 2.8% in the active treatment group (OR 0.21; 95% CI 0.11–0.42, p<0.0001). VKA, DOACs and aspirin significantly reduced the risk of recurrent VTE, with VKA and DOACs being significantly more effective than aspirin. Major bleeding events occurred in 12 patients in the control group (0.4%) and 25 of 3815 (0.6%) patients in the active treatment group (OR 1.64; 95% CI 0.69–3.90, NS). There were 39 (1.3%) deaths in control patients and 33 (0.9%) deaths in the anti-thrombotic group during the treatment period (OR 0.73; 95% CI 0.40–1.33, NS). Patients whose initial VTE event was a PE were more likely to have a recurrent PE than a DVT. The annualized event rate after discontinuation of extended antithrombotic therapy was 4.4% in the control group and 6.5% in the active treatment arm.

**Conclusions:**

VKA, DOACs and aspirin significantly reduced the risk of recurrent VTE, with DOACs and VKA being more effective than aspirin. The decision regarding life-long anticoagulation following an unprovoked DVT or PE should depend on the patients’ risk for recurrent PE as well as the patients’ values and preferences.

## Introduction

Venous thromboembolism (VTE), comprising deep vein thrombosis (DVT) and pulmonary embolism (PE) is a leading cause of patient morbidity and death.[1] TED may follow a definable provoking episode (most frequently hospitalization, surgery, trauma, malignancy or pregnancy) or may be unprovoked. Current guidelines recommend three months of anticoagulation to complete treatment of the acute episode of VTE (provoked or unprovoked); this is known as the “active treatment” phase.[2,3] Recurrent VTE after discontinuation of anticoagulation in patients with an idiopathic unprovoked DVT or PE occurs in between 20–30% patients followed for 10 years, with about 12% of recurrent events being fatal. [4–6] The risk of a recurrent event in patients who discontinue anticoagulation therapy after 3–6 months approximates 10% in the first year.[4–6] In the second year, the risk is estimated to be 5% and between 2–4% for each subsequent year.[4–6] Consequently, extending the period of anticoagulation beyond the initial 3 month period has been suggested in patients with unprovoked VTE; this is known as the “extended anticoagulation” phase. The risk factors for a recurrent event in patients with an idiopathic unprovoked VTE include male sex, increasing age and increasing body mass index.[4–7] Attempts to risk stratify patients into a low risk group who do not require extended anticoagulation based on patient demographics, the presence of a thrombophilia, and/or the d-dimer or repeat ultrasonography after stopping anticoagulation have generally met with limited success.[6,8–10] Testing for heritable thrombophilic defects is not useful for predicting recurrent events after a first episode of VTE.[11] Based on these data, the most recent clinical practice guidelines of the American College of Chest Physicians (ACCP) suggest extended anticoagulant therapy in patients with an unprovoked DVT or PE and who have a low or moderate risk of bleeding.[2] While anticoagulation is effective in preventing recurrent events in patients with unprovoked VTE the risk-benefit ratio and duration of extended anti-coagulation are uncertain. Furthermore, the risk of recurrent VTE after stopping extended anticoagulation and the role of aspirin and the direct oral anti-coagulants (DOACs) for secondary prophylaxis has not been clearly defined. Furthermore, there is a group of patients with VTE in whom the significance of the provoking event is unclear and where there is clinical equipoise regarding the continuation or cessation of anticoagulant therapy. The objective of this systematic review and meta-analysis was to determine the risk-benefit ratio of vitamin k antagonists (VKA), DOACs and aspirin in the secondary prevention of VTE and to determine the risk of recurrent events once extended anti-thrombotic therapy was stopped. While previous meta-analysis have addressed this issue,[12–14] they are limited by the fact that they included studies in which the initial period of anti-coagulation was less than 3 months, they included studies that did not include a placebo arm as well as studies that included patients with a second unprovoked VTE, were restricted to VKA’s only, evaluated low dose VKA (target INR 1.5–2), included a drug that has been discontinued for safety reasons (Ximelagatran),[15] and most importantly did not clearly distinguish between recurrent events that occurred during anti-thrombotic therapy and once therapy was stopped.

## Methods

### Identification of trials

Our aim was to identify all relevant randomized controlled trials that compared the risk of recurrent VTE in patients who had been treated for at least three months with a VKA or a DOAC for a first unprovoked DVT or PE and were then randomized to receive an oral anti-thrombotic agent or placebo for at least an additional six months. We included studies that included patients in whom clinical equipoise existed regarding the continuation or cessation of anticoagulant therapy. The anti-thrombotic agents we were interested in evaluating were VKAs, DOACs and aspirin. There was no restriction on language, publication year, setting or type of publication. Both authors independently searched the National Library of Medicine's MEDLINE database for relevant studies in any language published from 1966 to July 2015. The MEDLINE (OVID) search strategy is depicted in S1 Fig. In addition, we searched the Cochrane Register of Controlled Trial**s** (CENTRAL) and the bibliographies of all selected articles and review articles for other relevant articles. This search strategy was done iteratively, until no new potential citations were found. We performed and reported this meta-analysis according to the guidelines proposed by the PRISMA group. [16,17]

### Outcome variables

The primary outcomes were recurrent VTE (DVT or PE), major bleeding episodes and mortality during the treatment period. Only outcomes occurring during the time period that patients received study drug, placebo, or observation were included within the primary outcome analyses. Secondary outcome variable included the type of recurrent event (DVT or PE) and risk of recurrent VTE once active treatment had been stopped.

### Data extraction and quality assessment

Both reviewers independently assessed eligibility of articles identified in the initial search strategy for inclusion in the review. They discussed those papers deemed potentially eligible, independently extracted data using a standardized data abstraction form, and assessed studies’ methodological quality using the risk of bias assessment tool from the Cochrane Handbook for randomized trials.[18] Disagreements between the reviewers were resolved by consensus.

### Data analysis

Studies were grouped according to the type of antithrombotic agent (VKA, DOAC or aspirin). Summary data is presented as sample size, number of events and percentages as appropriate. We used standard meta-analytic techniques to summarize the data,[16,18] rather than a net-work meta-analysis model which combines direct and indirect estimates of effect in order to calculate the overall relative effectiveness of an intervention.[19] We used the random-effects model (more conservative) using Review Manager 5.3.4 (Cochrane Collaboration, Oxford) and considered p≤ 0.05 (two sided) as significant. We report binary outcomes as the odds ratio (OR). Summary effects estimates are presented with 95% confidence intervals (CI). We assessed heterogeneity between studies for each outcome using the Cochran Q statistic, [20] with p≤ 0.10 indicating significant heterogeneity,[21] and I^2^ with suggested thresholds for low (25–49%) moderate (50–74%) and high (>75%) values. We performed a Funnel plot to determine publication and study bias.[22–25]

## Results

**Fig 1:**
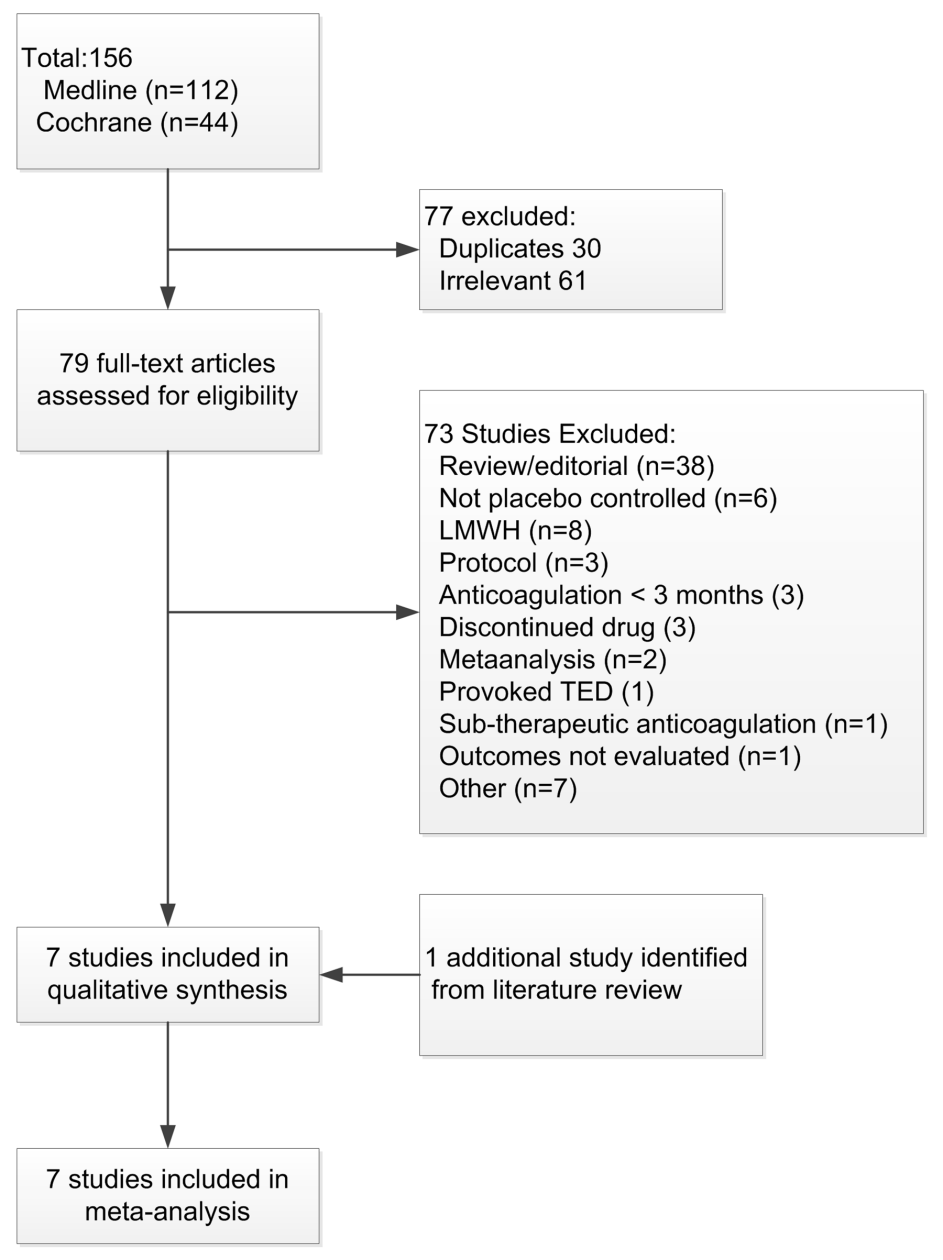
Flow-chart of studies evaluated for inclusion in meta-analysis.

**Fig 2:**
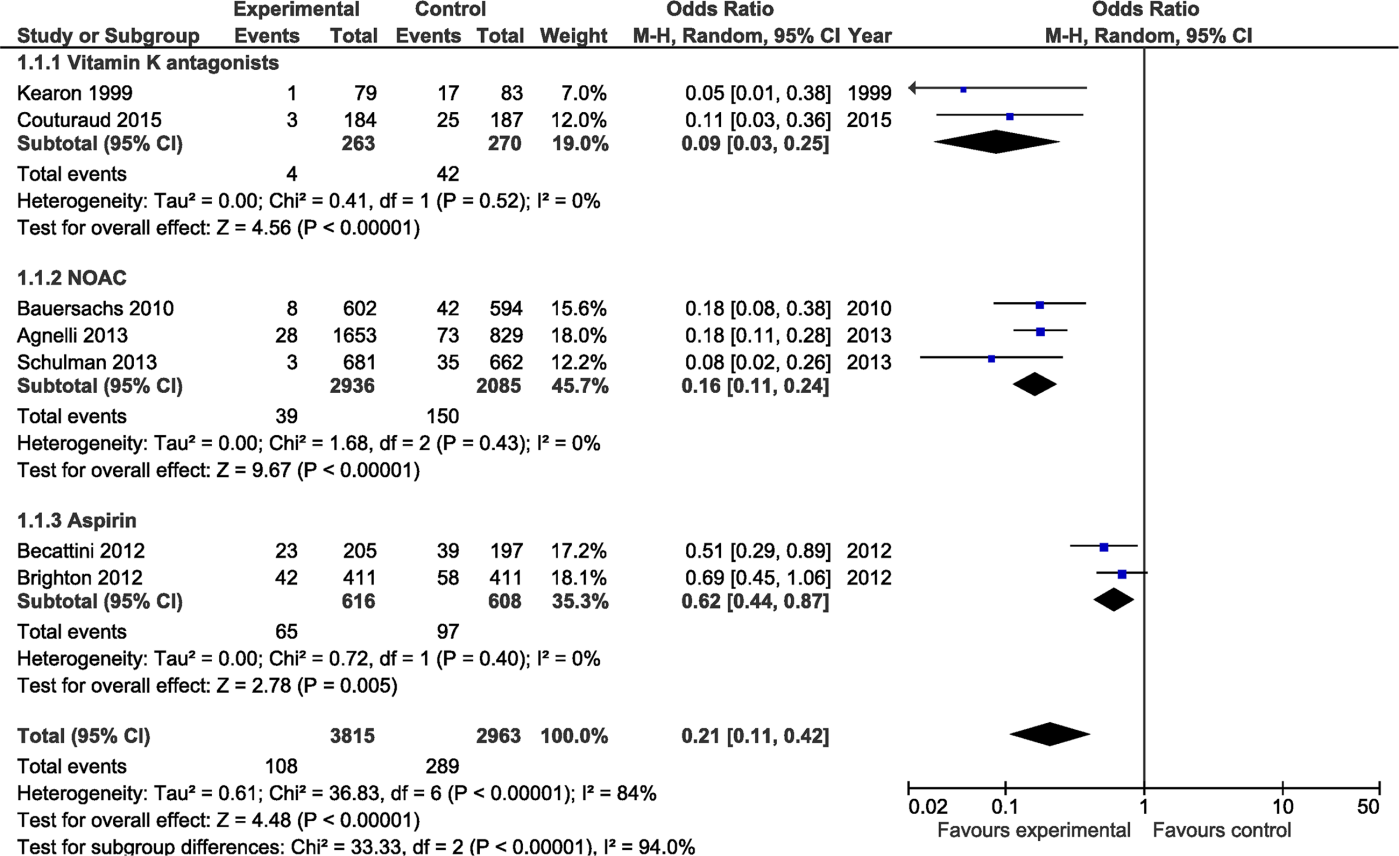
Comparison of the risk of recurrent thromboembolic events during the study period for patients receiving anticoagulant (intervention) versus control (placebo). Studies are grouped by type of anticoagulant. Weight is the relative contribution of each study to the overall treatment effect (odds risk ratio and 95% confidence interval) on a log scale assuming a random effects model.

Our initial search strategy identified 156 possible studies for inclusion in this analysis. The results of the Search strategy are depicted in Fig 1. Seventy-seven studies were excluded after screening and of the 79 full text articles assessed for eligibility73 were excluded. In addition to the six studies identified by our primary search [26–31], one additional papers was identified from a literature review.[32] The seven studies included in the systematic review and meta-analysis are summarized in Table 1. The risk of bias assessment of the included studies in depicted in S2 Fig. The Funnel plot for potential publication bias is depicted in S3 Fig. All the studies included were randomized, double-blinded, placebo controlled studies. Two studies evaluated the extended use of Coumadin, three studies evaluated a DOAC and two studies evaluated the use of aspirin. The apixaban study had three arms comparing a dose of 2.5 mg and 5 mg of apixaban with placebo.[30] As there was no difference in any outcome variable between the 2.5 mg and 5 mg dosages, [30] for the purposes of this meta-analysis we combined the data of the two dosage arms. In all 6778 patients were enrolled in these seven studies. The average sample size was 968 (162–2482) patients. In the Coumadin and aspirin studies 100% of the randomized patients had an unprovoked VTE. In the three DOAC studies between 73.5% and 93.2% of the VTE events were unprovoked; clinical equipoise with respect to continuation of anticoagulation existed in the remaining patients. The duration of the initial period of anticoagulation ranged from 3 to 18 months while the duration of extend anticoagulation ranged from six to 37 months (average 19.4 ± 11.7 months). In the control group 289 (9.7%) recurrent events occurred during the observation period compared to 108 (2.8%) events during treatment in the active treatment group (OR 0.21; 95% CI 0.11–0.42, p<0.0001); the annualized event rate was rate of 6.0% vs 1.7% respectively. VKA’s, DOACs and aspirin significantly reduced the risk of recurrent thromboembolic events, with VKAs and DOACs being significantly more effective than aspirin (no overlap of the 95% CI, see Fig 2.). All the studies except that of Couturand et al.[32] enrolled patients with both PE and DVT, with 64% of the events in these studies being a DVT. The nature of the recurrent event was recorded in three of these six studies, with 33% of the events being a PE. The study by Couturand et al included only patients with PE, with 82% of the recurrent events being a PE in this study.[32]

**Table 1:** Characteristics of studies included in meta-analysis.

Author	Year	Country	Agent	N	DVT/PE	Unprovoked (%)^a^	Duration Rx ^c^	PE ^d^	Follow- up ^e^	Rate Control ^f^	Rate intervention ^f^
**Kearon**	1999	Canada	Coumadin	162	TED (73% DVT)	100	3 vs 27	35	-	-	-
**Couturaud**	2015	France	Coumadin	371	PE	100	6 vs 24	82	24	3.7	6.7
**Brighton**	2012	Australia	ASA 100 mg	822	TED (57% DVT)	100	3–12 then 37	-	-	-	-
**Becatlini**	2012	Italy	ASA 100 mg	402	TED (63% DVT)	100	6–18 then 24	-	-	-	-
**Bauersachs**	2010	Multiple	Rivaroxaban	1196	TED (62% DVT)	73.5	6–12 then 6–12	28	1		
**Agnelli**	2013	Multiple	Apixaban ^b^	2482	TED (65% DVT)	91.6	6–12 then 12	-	-	-	-
**Schulman**	2013	Multiple	Dabigatran	1343	TED (64% DVT)	93.2	6–18 then 6	37	12	5.1	6.4

^a^ Patients without a clearly definable provoking factor

^b^ 2.5 or 5 mg BID

^c^ Months

^d^ Percentage of recurrent thromboembolic events that are PE

^e^ Months of follow-up after stopping anticoagulation

^f^ Event rate after stopping intervention (percentage events/year)

Abbreviations: DVT—deep venous thrombosis, PE—pulmonary embolus, TED—thromboembolic disease, Rx—therapy

**Fig 3:**
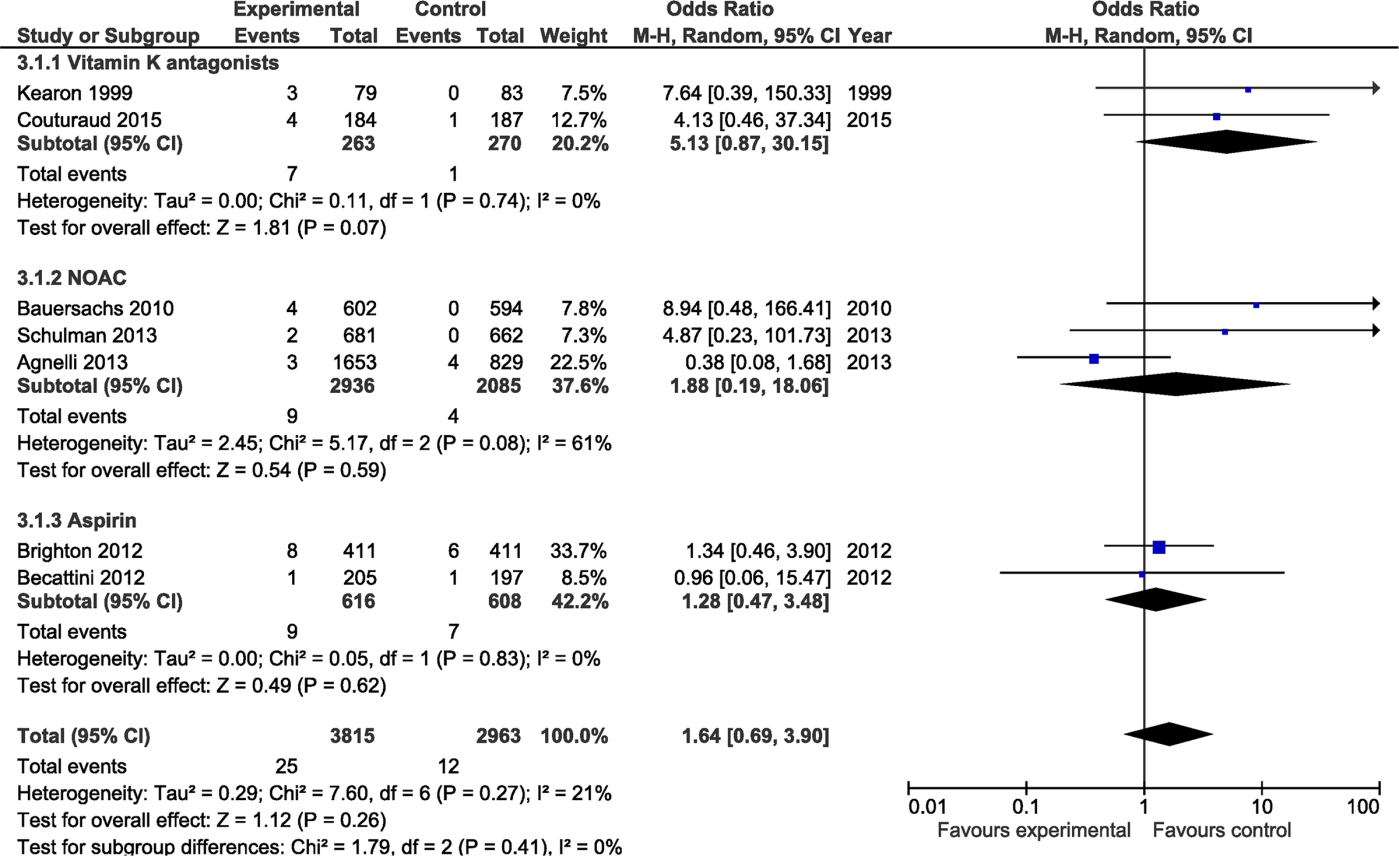
Comparison of the risk of major bleeds during study period for patients receiving anticoagulant (intervention) versus control (placebo). Studies are grouped by type of anticoagulant. Weight is the relative contribution of each study to the overall treatment effect (odds risk ratio and 95% confidence interval) on a log scale assuming a random effects model.

**Fig 4:**
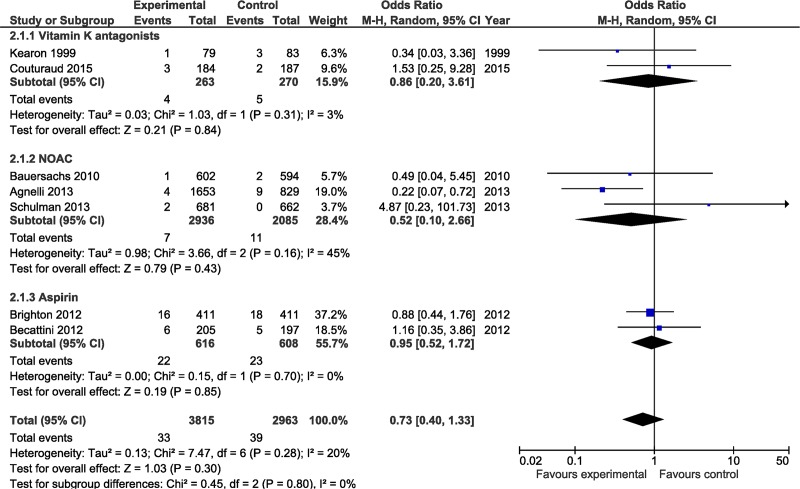
Comparison of the risk of mortality during study period for patients receiving anticoagulant (intervention) versus control (placebo). Studies are grouped by type of anticoagulant. Weight is the relative contribution of each study to the overall treatment effect (odds risk ratio and 95% confidence interval) on a log scale assuming a random effects model.

There was a trend towards an increased risk of major bleeding in the active treatment group, however this did not reach statistical significance (OR 1.64; 95% CI 0.69–3.90, NS) (See Fig 3). Major bleeding events occurred in 12 patients in the control group (0.4%) as compared to 25 of 3815 (0.6%) patients in the active treatment group. Overall there were 39 (1.3%) deaths in control patients as compared to 33 (0.9%) deaths in the anti-thrombotic group during the active treatment period (OR 0.73; 95% CI 0.40–1.33, NS) (See Fig 4). The event rate following discontinuation of active treatment (in both the control and treatment arm) was determined in 2 studies with a duration of followup of 12 and 24 months (one study followed patients for 1 month following discontinuation) (see Table 1). The annualized event rate was 4.4% in the control group and 6.5% in the active treatment arm.

## Discussion

The results of our meta-analysis demonstrated that VKAs, DOACs and aspirin significantly reduced the rate of recurrent VTE events in patients treated for up to 37 months, with the efficacy of VKA’s and DOACs being equivalent and more effective than aspirin. The number of major bleeds and deaths was low in all three groups; this meta-analysis was therefore underpowered to determine a difference for these outcomes. It should however be recognized that the studies were heterogeneous with respect to duration of the initial period of anticoagulation, duration of extended anticoagulation and characteristics of the study population. Despite this heterogeneity the benefit was consistent amongst all the studies.

The current ACCP guidelines suggest that patients who have suffered a first unprovoked VTE should be considered for “indefinite” anticoagulation.[2] No trial has randomized patients with VTE to stop or continue anticoagulants and then followed patients indefinitely (e.g, for 10 or more years).[3] The trials included in this analysis followed patients receiving extended anticoagulation for between 6 months and 3 years. Three trials followed patients once the extended anticoagulation was stopped; these studies demonstrated an average annualized event rate of approximately 5% in both patients who received extended anticoagulation and placebo. This rate is similar to the annual event rate in patients with an unprovoked VTE treated for 3–6 months with anticoagulation who were then followed for up to 10 years. [4–6] This suggests that patients with an unprovoked VTE, much like patients with atrial fibrillation, have a life-long increased risk of thromboembolic complications. Furthermore, the benefit of anticoagulation continues only for as long as therapy is continued.[3] Consequently, long-term anticoagulation may, in effect, equate to lifelong treatment, or for as long as the perceived risk of anticoagulant therapy-related bleeding is not so high as to preclude continued treatment.[3]

While life-long anticoagulation will reduce the risk of recurrent VTE the risk-benefit ratio is not equal amongst all patients. The major reason to treat patients lifelong with anti-coagulation is to prevent fatal PE and recurrent PE leading to chronic thrombo-embolism and pulmonary hypertension. However, recurrent DVT increases the risk of developing the post-thrombotic syndrome.[33] For reasons that are somewhat obscure patients with a previous PE are at much higher risk of recurrent PE than are patients whose primary event was a DVT. Agnelli et al randomized patients with DVT to extended anticoagulation or to a control group. [34] In this study only 14% of recurrent events were PE. This contrasts with the study by Couturaud and colleagues, who randomized patients with only PE to extended anticoagulation or placebo.[32] In this study 82% of recurrent events were PE. In the study conducted by Brighton et al,[28] PE occurred in 30% of patients with previous DVT and 73% in those with a previous PE. In a patient level meta-analysis of 2554 patients with VTE, Baglin et al reported a 5 year cumulative recurrent rate of 22.6%; the recurrence rate was similar in patients presenting with symptomatic PE compared to symptomatic DVT alone.[35] In the patients with proximal DVT alone, the cumulative rate of recurrence as PE was 3.8%. However, in the patients who initially presented with a PE, almost 50% of the recurrent events were a PE. This data suggests that whilst DVT and PE are considered manifestations of the same pathology, the phenotypic expression of the disease appears to be predetermined. This data suggests that the balance of benefit and risk of long-term anticoagulation is different in patients whose initial event is either a PE or DVT. In patients whose initial event is an unprovoked PE (or when the significance of the provoking event is unclear) life-long anticoagulation may be the best option even if the patient is considered to be at an increased risk of bleeding. In patients whose initial event is a DVT the risk-benefit ratio of long term anticoagulation should be made on an individual basis taking into consideration the patients’ risk profile as well as their values and preferences. Three clinical prediction rules have been developed to estimate the risk of recurrence in patients with unprovoked VTE. They take into account, with some differences, combinations of sex, D-dimer levels, site of initial thrombosis, age when VTE occurred, and signs of post thrombotic syndrome (1 rule).[6,36,37] The ability to predict the risk of recurrence, and to improve patient outcomes, has yet to be prospectively demonstrated for these rules.[3]

Patients who are treated indefinitely should be reviewed by their physicians at least annually to ensure that the continued benefits of anticoagulation outweigh the risks, that they have not developed a contraindication to anticoagulant therapy, that their preferences have not changed, that precautions have been taken to limit the risk of bleeding (blood pressure control avoidance of other anti-thrombotic agents) and that they are being treated with the most suitable anticoagulant regimen.[3]

The meta-analysis reported here combines data across studies in order to estimate treatment effects with more precision than is possible in a single study.[16] The main limitation of this meta-analysis is that the patient population and the duration of the active treatment and extended treatment phases were not the same across studies. In addition, the three DOAC studies included patients with “provoked” VTE in whom clinical equipoise with respect to continuation of anticoagulation existed. These studies did not stratify outcomes according to whether the provoking event was provoked or unprovoked. Furthermore, the duration of extended anticoagulation of the studies included averaged only 18.5 months precluding making strong recommendations with regards to the risk and benefits of lifelong therapy. Nevertheless, we believe that the quality of the studies included and the robustness of the analysis allow clinicians and their patients to make informed decisions regarding extended anticoagulation for the secondary prevention of idiopathic VTE. An additional potential limitation of our meta-analysis is that our search strategy was limited to the MEDLINE and CENTRAL databases and we did not search the EMBASE, LILACS and Euro-Pubmed databases. Furthermore, we did not include the term “acenocoumarol” in our search strategy (S1 Fig). However, the bibliographies of all selected articles and review articles were closely reviewed for other relevant articles, making it unlikely that additional relevant studies were missed.

## Conclusions

Patients with an unprovoked initial DVT or PE and those in whom clinical equipoise exists with regards continuation of anticoagulation are at a substantial risk of having a recurrent VTE event after the initial active treatment phase. VKAs, DOACs and aspirin all reduce the risk of recurrent VTE, with VKA’s and DOACs being more effective than aspirin. Not all patients may benefit equally from lifelong anticoagulation and this decision should be based on the inciting event (DVT or PE) as well as the patients’ risk profile together with the patients’ preferences.

## Supporting Information

S1 Fig Medline (OVID) search strategy (TIF)

S2 Fig Risk of bias assessment. (TIF)

S3 Fig Funnel plot for potential publication bias for studies comparing anti-coagulation to placebo/control. SE, standard error; OR, odds ratio.
(TIF)

S1 Table PRISMA Checklist. (DOC)
