# Supplementary figures and images for: Extended Anticoagulant and Aspirin Treatment for the Secondary Prevention of Thromboembolic Disease: A Systematic Review and Meta-Analysis
Source: PLoS One. 2015 Nov 20;10(11):e0143252. doi: 10.1371/journal.pone.0143252 (PMC4654552; doi:10.1371/journal.pone.0143252)

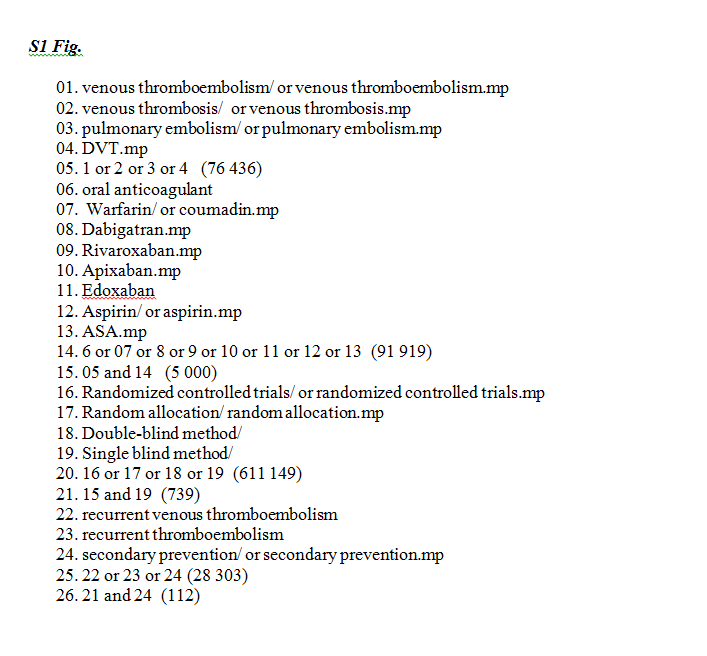

Supplement: S1 Fig — (TIF) [file pone.0143252.s001.tif]

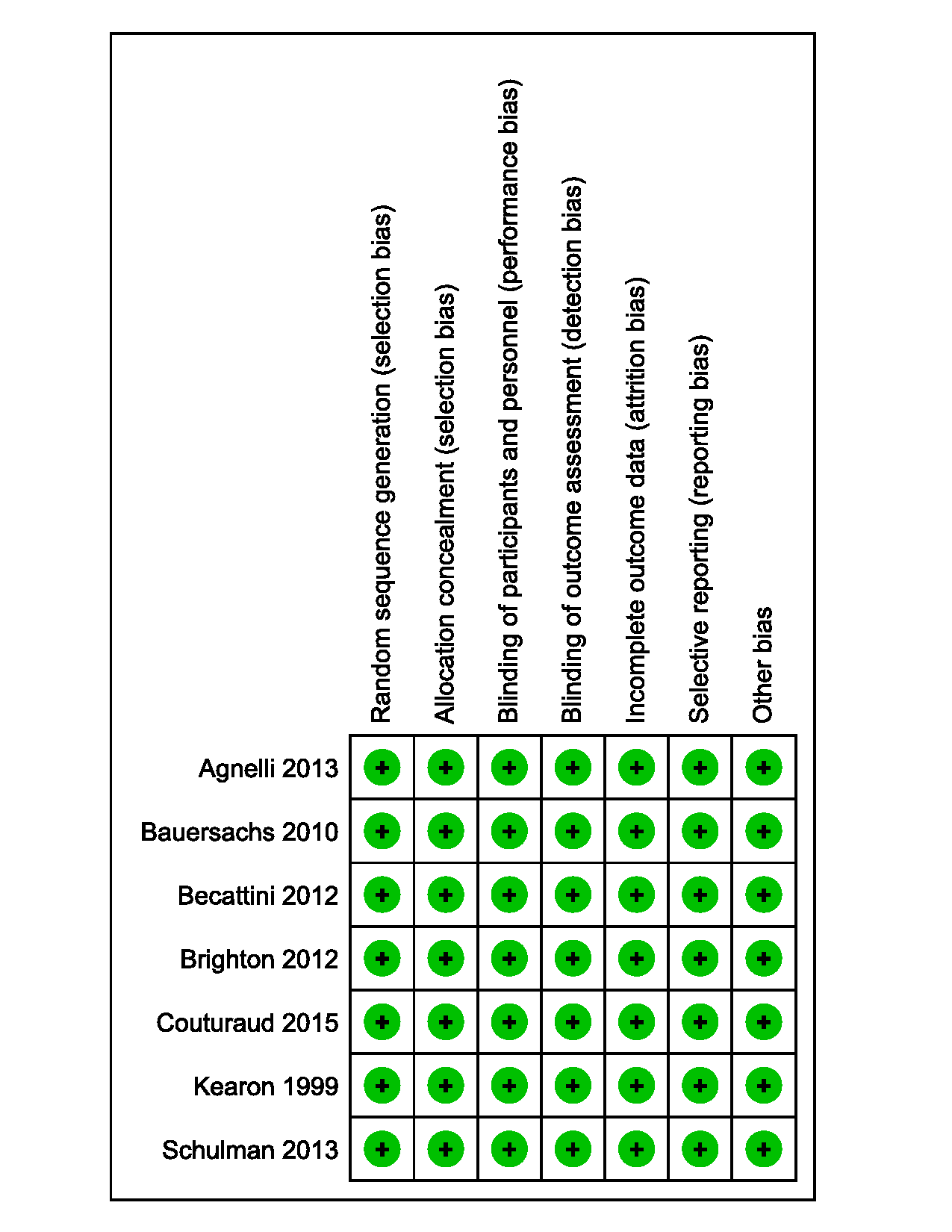

Supplement: S2 Fig — (TIF) [file pone.0143252.s002.tif]

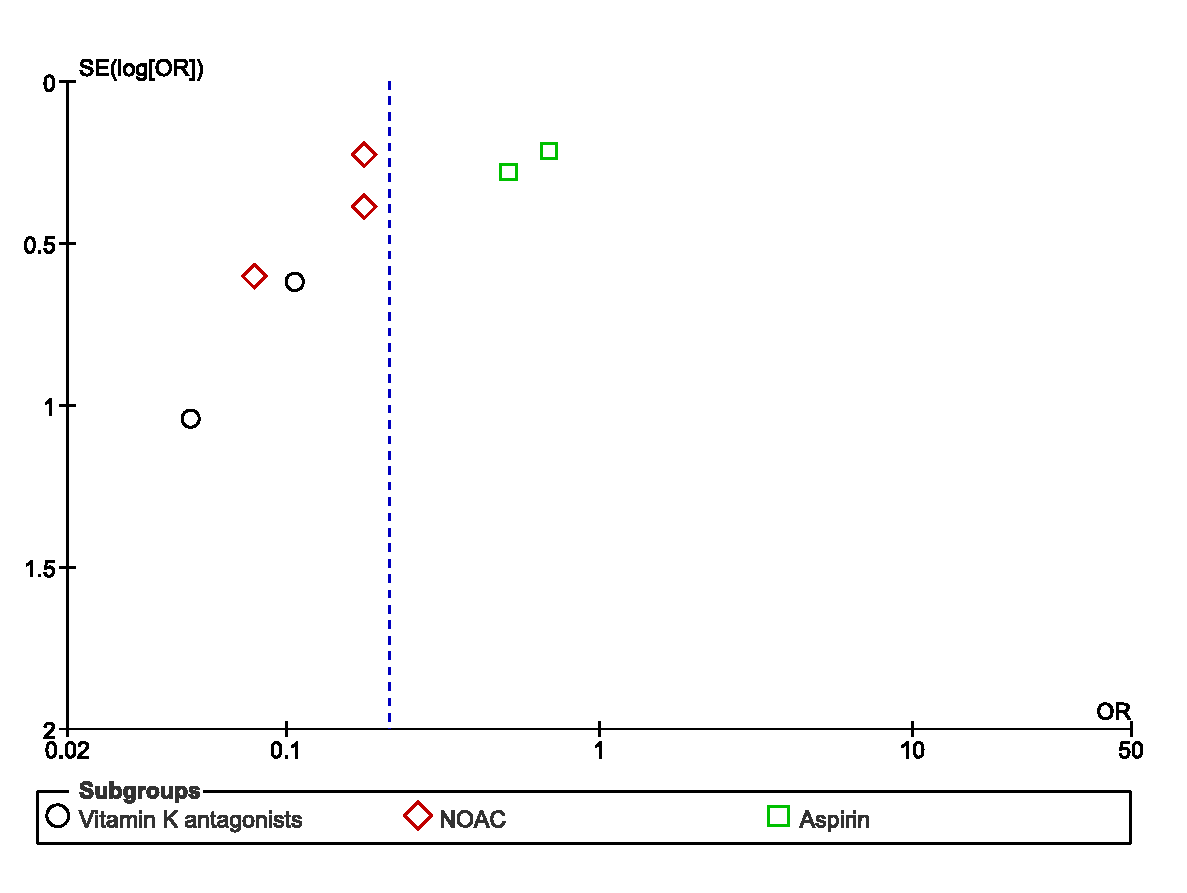

Supplement: S3 Fig — SE, standard error; OR, odds ratio. (TIF) [file pone.0143252.s003.tif]
